# Physiological Uterine Involution in Primiparous and Multiparous Women: Ultrasound Study

**DOI:** 10.1155/2017/6739345

**Published:** 2017-05-07

**Authors:** V. Paliulyte, G. S. Drasutiene, D. Ramasauskaite, D. Bartkeviciene, J. Zakareviciene, J. Kurmanavicius

**Affiliations:** ^1^Clinic of Obstetrics and Gynaecology of Vilnius University, Centre of Obstetrics and Gynecology, Vilnius University Hospital Santariskiu Klinikos, Santariskiu 2, LT-08661 Vilnius, Lithuania; ^2^Department of Obstetrics, University Hospital Zurich, Zurich, Switzerland

## Abstract

**Purpose:**

To examine the uterine involution period after uncomplicated delivery in primiparous and multiparous women.

**Methods:**

Longitudinal prospective study. Repeated parameters were measured and endometrial contents and diastolic notch were observed. Measurements of primiparous and multiparous women were carried out after labour on the 1st, 3rd, 10th, 30th, 42nd, and 60th postpartum days. The analysis was performed using SPSS version 21.

**Results:**

The median uterus parameters are bigger in multiparous group in physiological puerperium, but the decreasing trend is the same. The endometrial cavity on the 10th day was significantly wider in multiparous women and mainly echo-negative view of the uterine cavity was observed. The evaluation of the uterine angle deviation changes from an extremely retroverted position to a more anteverted position. RI of the uterine artery in both groups was low immediately after labour and significantly increased one month postpartum. Notching of the uterine artery undergoes changes, but diastolic notch does not appear in all postpartum women even after two months following labour.

**Conclusions:**

The puerperium period after normal vaginal delivery depends on parity. The trend of involution in primiparous and multiparous women follows a similar pattern, yet, it lasts longer in the multiparous women. Ultrasound of uterine is certainly a useful tool after labour and may be important in facilitating an early detection of postpartum uterine complications.

## 1. Introduction

The physiological puerperium period is still not fully investigated. A number of ultrasound studies focus on puerperium and describe the changes detected in the size, the shape, the position, and the texture of the uterus [1–5]. Most of them report on the normal involution period of 6 weeks following labour after normal or pathological delivery, without addressing the differences in parity [1–8]. There is still a shortage of studies describing the uterine ultrasound differences found in primiparous and multiparous patients after normal labour from the earliest puerperium until 8 weeks of postpartum period [9]. Only a few studies include Doppler measurements of uterine arteries during the normal involution period, or the scope of the examination is very narrow [1, 3, 10–14]. A longitudinal sonographic study is the best way to explore the similarities and differences that are likely to occur in primiparous and multiparous patients during puerperium. The aim of this study is to compare the metrical quantitative and the qualitative characteristics of the uterus in the puerperium in primiparous and multiparous women after normal vaginal delivery.

## 2. Methods

A longitudinal prospective study was carried out in Vilnius University Hospital Santariskiu Klinikos. 64 participants were invited to this study at the first stage of singleton term delivery. The inclusion criteria were as follows: older than 18 years of age, conscious women, without any mental disorders or congenital diseases, after vaginal term delivery. The exclusion criteria were as follows: preterm or multiple pregnancy, stillbirth, complicated postpartum bleeding (intensive care measures were used, with B-Lynch suture after labour and uterine devascularisation), congenital uterine disorders (bicornuate or unicornuate uterus, double uterus), uterine fibroids or oncological diseases, uterine scar, retained placental tissue, or endometritis postpartum. 18 women were excluded from this study because of later appearing rejection criteria (postpartum bleeding, retained products of conception, placenta accreta, caesarean section, endometritis postpartum, and hysterectomy after labour); these ladies were observed as pathological group and data of them will not be presented in this study. Of the 46 women included in this study, 24 were primiparous (group I) and 22 multiparous (group II). A serial ultrasonographic examination was carried out on the 1st, 3rd, 10th, 30th, 42nd, and 60th days of the postpartum period. The first examination was performed within two hours after delivery. Each woman was examined 6 times, with the exception of 4 missed exams in group I and 2 exams in group II (for personal reasons). An abdominal ultrasound scan was carried out on the the 1st, 3rd, and 10th days, while transvaginal sonoscopy was carried out, on the 30th, 42nd, and 60th days. GE Healthcare Voluson S6 and Voluson S8 systems were used to evaluate gray-scale, colour and pulse Doppler ultrasound measurements. Uterine measurements were performed on the basis of commonly used recommendations for pelvic ultrasound and Doppler scans [15–22]. All the participants were provided with oral and written information about the study. The study was approved by the Regional Bioethics Committee of the Faculty of Medicine of Vilnius University (number 158200-13-605-183).

**Figure 1:**
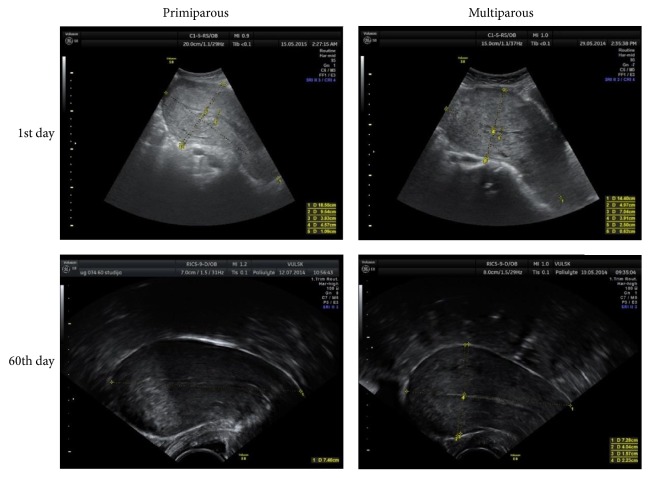
Uterus length measurements in longitudinal section.

**Figure 2:**
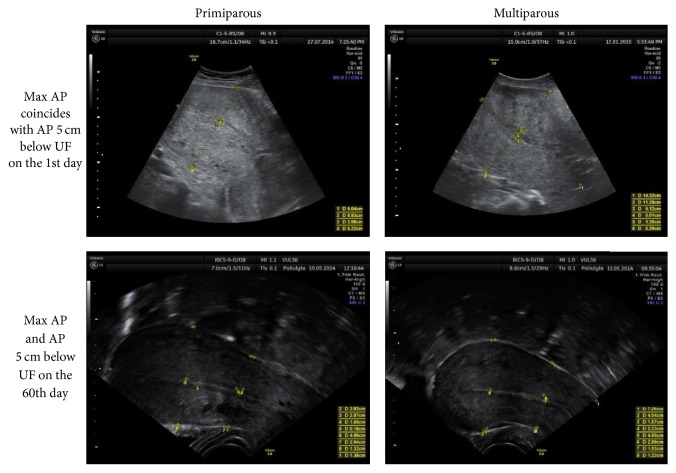
Max AP and AP 5 cm below UF measurements in longitudinal section.

The uterine length (Figure 1) and the anteroposterior diameter (AP) (Figure 2) were measured in longitudinal sections. The AP diameter was measured in two points: in the widest part of the longitudinal section and 5 cm below the uterine fundus (UF), perpendicular to the longitudinal uterine axis. As usual, in praxis, the measurements are performed in the widest (maximum) part of the uterus [3–5, 15–20]; however, some researchers suggest measuring 5 cm below the uterine fundus [1, 6–8]. This study compares both points of measurement in primiparous and multiparous patients.

**Figure 3:**
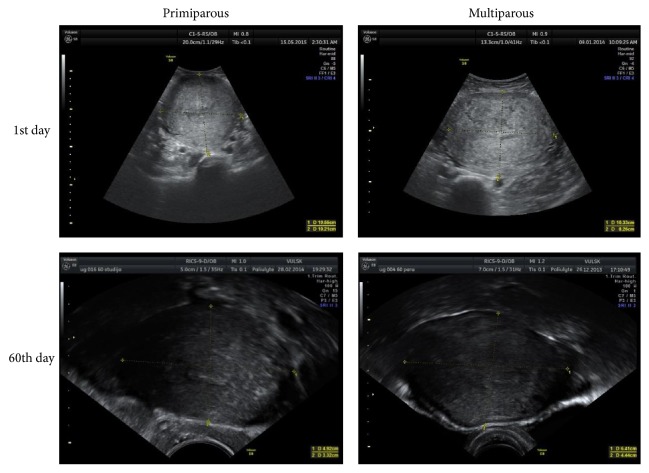
Uterus width measurements in transverse section.

**Figure 4:**
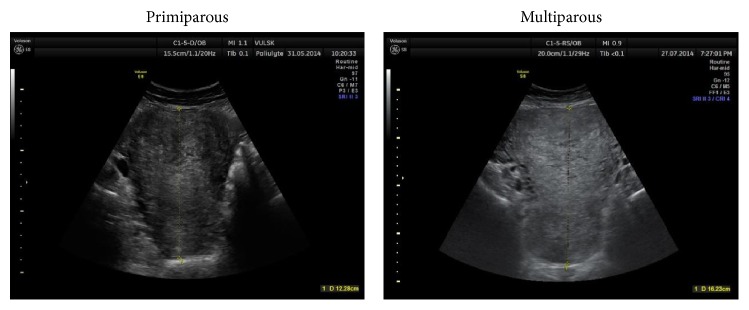
Coronal view of uterus was measured on the 1st day (within two hours after labour).

The uterine width was measured in transverse section (Figure 3), the coronal view was evaluated on the 1st day to exclude congenital malformations of the uterus (Figure 4).

**Figure 5:**
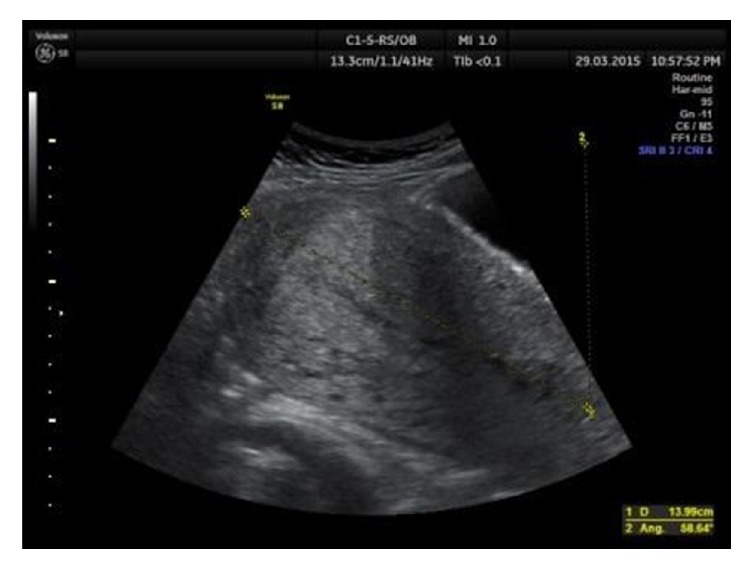
Uterine angle (in degrees) measurement in relation to the longitudinal axis of the body.

The endometrial stripe thickness and the endometrial contents were evaluated in a longitudinal section. The uterine angle was measured in relationship with the longitudinal axis of the body (Figure 5).

**Figure 6:**
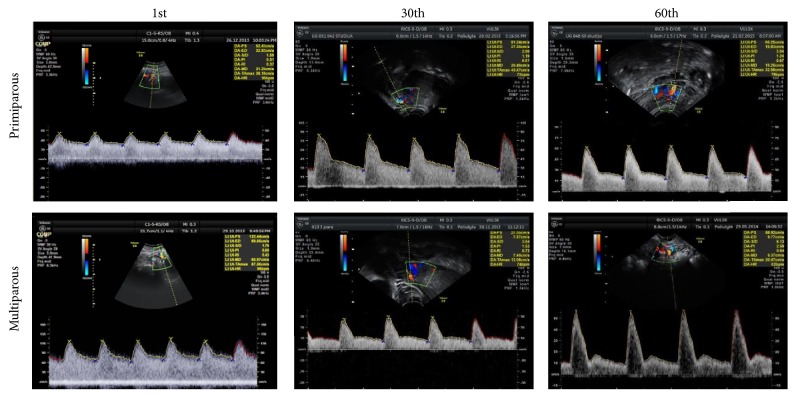
Uterine artery flow changes on the 1st, 30th, and 60th days in primiparous and multiparous women.

The colour and Doppler measurements of the uterine artery (resistance index (RI = (PSV − EDV)/PSV = (peak systolic velocity − end diastolic velocity)/peak systolic velocity)) were performed at the point where this artery crosses the external iliac artery (beam/flow angles were kept at 30°) [17–23]. The mean values of both left and right arteries were used (Figure 6).

In this study were compered also some other parameters: mother's age, BMI, anemia, B group streptococcus infection, smoking, duration of labour and the anhydrous period, infant birth weight, meconium stained amniotic fluid, placental site (anterior or posterior uterine wall), induction of labour, use of Oxytocin for labour augmentation, and breastfeeding duration.

All the analyses were performed using SPSS, version 21. Continuous variables were summarized using descriptive statistics, including the number of subjects, mean, standard deviation, median, and confidence intervals with minimum and maximum values. Kruskal–Wallis test was used to evaluate the associations between primiparous/multiparous women and uterine parameters expressed as continuous variables. Categorical variables were expressed as numbers and percentages. Chi-square test was used to examine the relationships between primiparous/multiparous women and other categorical variables.

## 3. Results

24 primiparous and 22 multiparous women, who underwent uncomplicated vaginal delivery and none of whom suffered from any complications (fever or hemorrhage) during puerperium, were examined after delivery from 2013 through 2016 (the characteristics of these women are presented in Table 1).

The size of the uterus was measured by the length of the uterus, the uterine width, and the AP diameter. Almost all these parameters are dependent on parity. The uterus is slightly larger in multiparous women within two hours after childbirth and these indicators maintain higher values to the end of puerperium (Table 2). The size of the uterus decreases rapidly over the first 30 postpartum days (1st, 3rd, 10th, and 30th days); later, the involution decreases steadily till two months postpartum.

**Figure 7:**
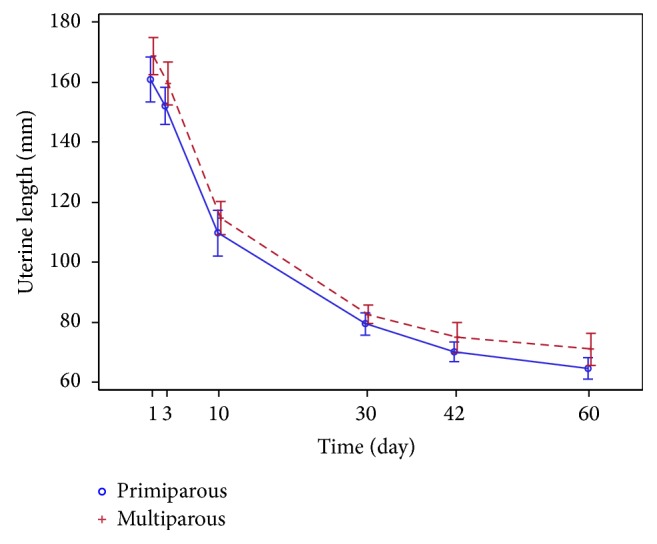
Uterine length (mm) regression.

**Figure 8:**
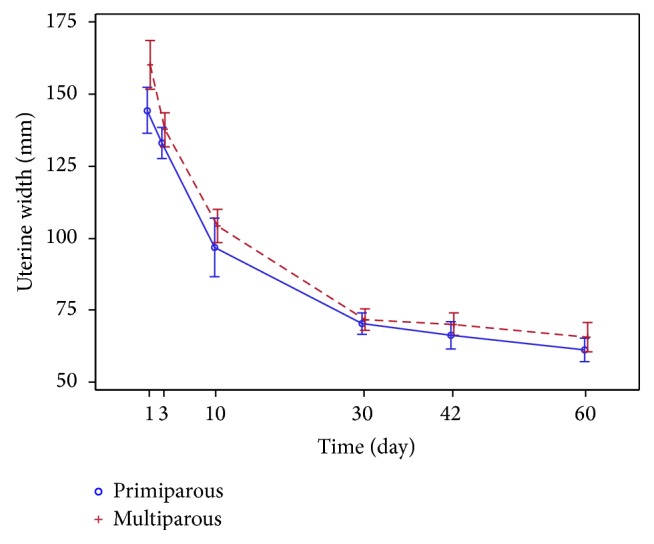
Uterine width (mm) regression.

The trends of regression in the uterine dimensions* (the length, the width, and the AP diameter)* observed over two months after childbirth are similar in both groups (Figures 7 and 8).

**Figure 9:**
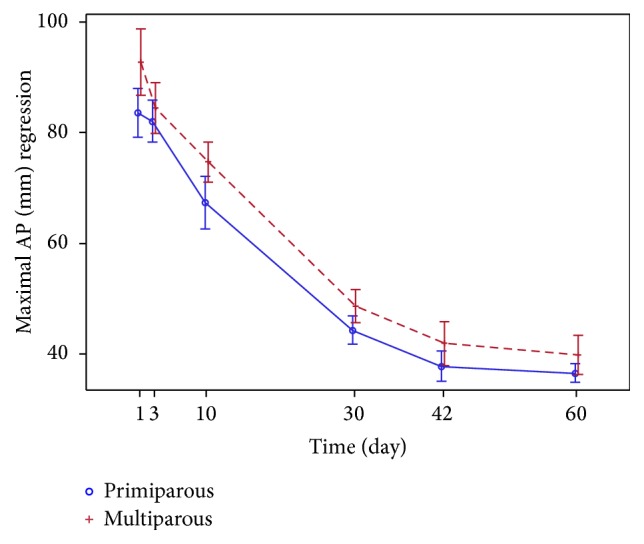
Maximal AP (mm) regression.

**Figure 10:**
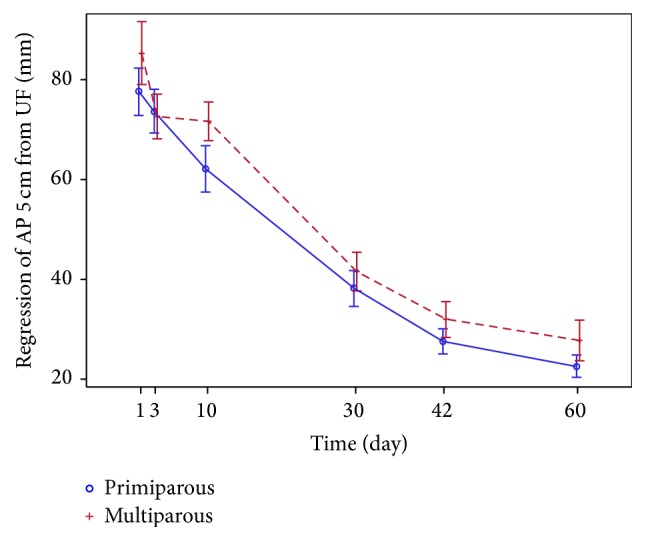
Regression of AP 5 cm below UF (mm).

*The AP diameter* is higher for the multiparous subjects in both points of measurement (maximal and 5 cm below UF) on the 1st day postpartum and remains larger on the 60th day. AP decreases following the same pattern as with other parameters of the uterus during the entire involution period in both points of both the groups (Figures 9 and 10). On the 1st day, maximal AP most commonly coincides with AP 5 cm below UF, but never at the end of the uterus involution period (Figure 2).

**Figure 11:**
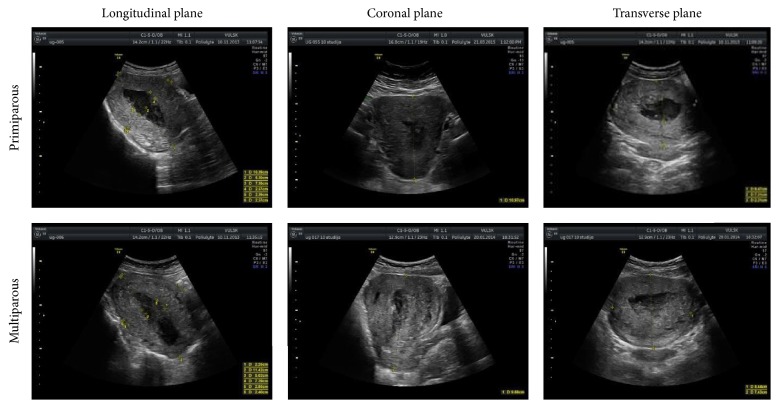
Most frequent uterine cavity inserts in all planes on the 10th day (primiparous and multiparous).

The 10th postpartum day is a special time for the uterine involution given the occurrence of dramatic changes in the uterine cavity during the normal puerperium experienced by both groups of women. It is important to assess these changes in terms of uterine physiology. Mostly a wide echo-negative view of the uterine cavity in all the planes of the uterus is observed (64% for both groups), less frequent, mixed view (22% primiparous and 36% multiparous), while an echo-positive view is rare (14% primiparous and 0% multiparous) (Figure 11).

The differences found in the endometrial cavity changes over the uterus involution period between primiparous and multiparous subjects showed no statistically significant difference; however, the difference observed on the 10th postpartum day was statistically significant (in primiparous women 9.5 ± 9.9 mm, in multiparous women 18.5 ± 7.2 mm, *p* = 0.001) (Table 3).

The uterine angle deviation, in relation to the longitudinal axis of the body, changes from a particularly retroverted position to a more anteverted one. The differences found between primiparous and multiparous women are not statistically significant, but the angle changes are likely to be increasingly larger during puerperium in multiparous women (median difference from the first to the 60th day is 94.0 ± 64.1 degrees in multiparous women and 21.5 ± 66.7 degrees in primiparous women (*p* = 0.0005)).

**Figure 12:**
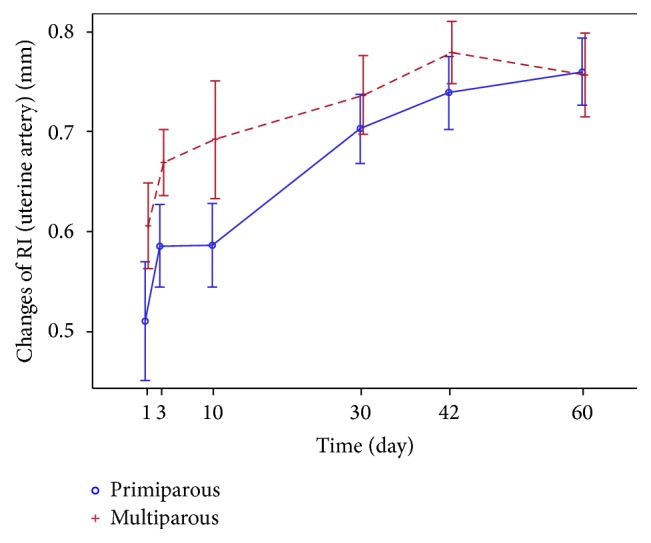
Changes of RI (uterine artery) in primiparous and multiparous women.

The uterine artery flow examination and index (RI) measurements showed significant changes in both groups until midpuerperium.* The resistance* (RI) of the uterine artery was low immediately after childbirth and showed a significant increase one month after parturition in both groups (Figure 12); later, these changes tend to be more steady.

The largest RI difference recorded in primiparous and multiparous women was within the first 10 postpartum days, while at the end of puerperium, no resistance differences were recorded (Table 4).

**Figure 13:**
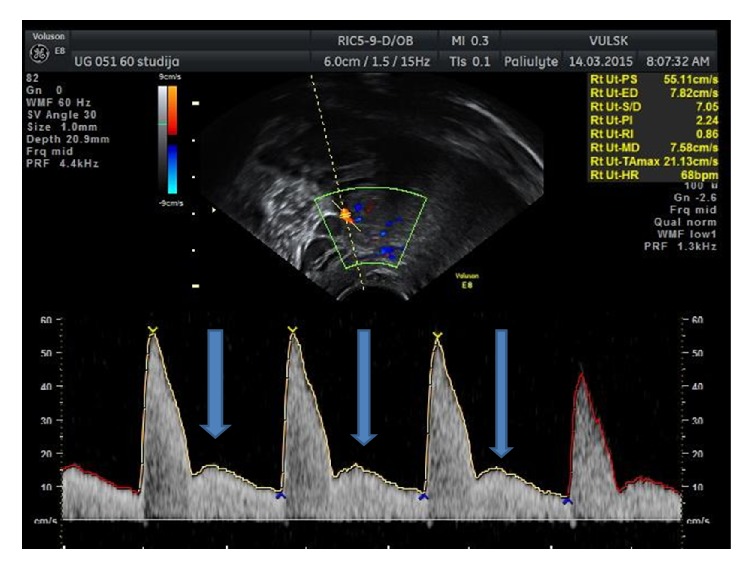
Notching of the uterine artery.

**Figure 14:**
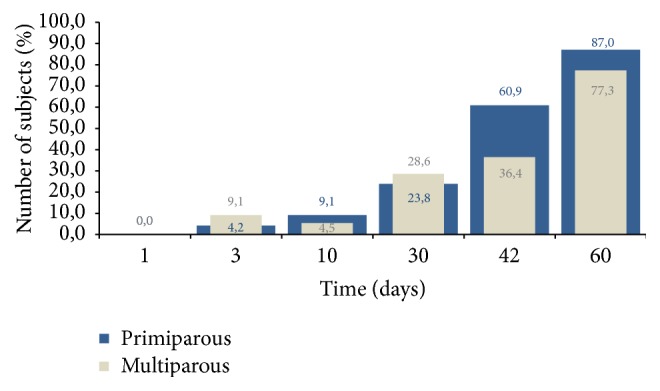
Diastolic notch appearance during puerperium.

Notching of the uterine artery (Figure 13) undergoes changes during puerperium; however, the appearance of the diastolic notch is observed not in all women even after two postpartum months (Figure 14).

In this study, an attempt was also made to find correlations between the normal puerperium of primiparous and multiparous women and maternal parameters such as mother's age, BMI, anemia, B group streptococcus infection, smoking, duration of labour and the anhydrous period, infant birth weight, meconium stained amniotic fluid, placental site (anterior or posterior uterine wall), induction of labour, use of Oxytocin for labour augmentation, and breastfeeding. Unfortunately, no relevant correlations were found.

## 4. Discussion

The uterine involution starts immediately after the delivery of placenta [24]. Understanding of normal view of the uterus during the entire period of puerperium helps practitioners to avoid unnecessary interventions for alleged retained products of conception (RPOC) or atonic uterus [6–8, 16]. During the normal puerperium period, the uterine involution is defined by the changing indices of the uterine size, the uterine cavity inserts, and the uterine artery flow [1–5, 15]. Until recently, there were no studies showing a view of the uterus immediately after childbirth. Most of the studies publish the first ultrasound examination findings on the 1st, 2nd, and 3rd postpartum days [1, 4, 11–13], but there is not a single ultrasound study examining the uterus within the first two hours after delivery. The strengths of this study are as follows: the research, from the beginning to the end, was conducted by one person; the same person assisted the women under analysis during delivery; the first data are obtained from the earliest puerperium (within two hours after delivery); a detailed explanation of the differences observed between primiparous and multiparous women is provided. The information obtained from the findings of this study on the uterus view over this period is highly efficient in postpartum hemorrhage cases. Nowadays, the doctor can bring a portable ultrasound machine to the delivery room and examine the uterus for RPOC. If we see no RPOC, we can use conservative measures for treatment without any interventions. The knowledge acquired on the physiological differences occurring between primiparous and multiparous females over the puerperium period facilitates differentiating a normal uterine contraction from an inadequate one in case of atonic uterus [1, 24]. The findings of this study showed that although the multiparous uterus shrinks more intensively [25, 26], it still remains of a larger size from the very early till the late puerperium.

Most of the authors [1–5], except for one who represents the newest studies [9], show no correlation between the involution of the uterus and parity. This study shows the differences observed in the uterus size of primiparous and multiparous women. Statistically significant bigger AP and uterus* width* in multiparous than primiparous women were found within one month after childbirth. Other parameters revealed that the uterine size tends to be larger in the multiparous, yet no significant differences were found. Even though the points/sites of the AP measurement can be considered the most debatable issue in this study, we compared the measurements conducted in two points: in the widest part and 5 cm below UF of the longitudinal uterine view. We recommend that AP is measured in the widest part of the longitudinal view of the uterus in the same way a nonpregnant uterus is measured [3–5, 15–20]. The AP measurements 5 cm below UF are perhaps more defined; however, they will be inaccurate at the end of puerperium (they may even occur at the cervical part of the uterus) (Figure 2).

This study is intended to draw attention to the 10th day, when the diagnosis of the retained products of conception (RPOC) could be made by mistake due to a special view of the uterine cavity. All of the women involved in the study (both groups) complained of the increased vaginal bleeding on the 10th–14th postpartum days, especially after physical exertion or more frequent breastfeeding, and the ultrasound findings show mostly fluid insertion of the uterine cavity in both groups at this period. The same trend was found by other authors [1, 4, 5]; however, they did not find any correlation between the uterine cavity and parity. Our study found statistically significant larger width of uterine cavity in multiparous women. Thus, this period of involution should be kept in mind by practitioners seeking to distinguish the physiological and pathological changes, especially in multiparous women.

With reference to the results obtained from our study and supported by other authors, the RI of the uterine artery between the 3rd and 10th postpartum days showed a slight increase, while at the end of the 1st-month postpartum, it increased significantly. In both groups of women, these indices are continuously increasing over a period of time from the 30th till the 42nd day (Figure 12) and remain stable from 6 till to 8 weeks after labour, in contrast with other uterine parameters that are continuously changing (uterine length, width, and AP diameter) [1, 7, 12–14, 22]. Unlike many authors, this study found statistically significant differences between primiparous and multiparous women taking into account the uterine artery flow indices at the first two postpartum hours, yet, opposite to Guedes-Martins et al. [9], we found a higher uterine artery RI in the multiparous than in the primiparous group during early puerperium. At the end of the puerperium period, the RI data is almost identical in both groups.

Notching of the uterine artery is one of the indices of the uterine involution changes [1, 2, 6, 7, 13, 14] during puerperium, but an absent diastolic notch cannot be a negative indicator of involution, because even two months after labour a diastolic notch does not appear in all women (Figure 14) [27]. On the 1st day (within two hours after labour), the diastolic notch was absent in all of the observed women from both groups; some authors count 13–22.5% of notching in the first week after childbirth, but no one suggested any evidence on notching within two hours postpartum [1–3, 7, 9]. We found a more frequent notching in multiparous women on the 3rd and 30th days; however, on other days, notching appearance is higher in primiparous women. Our findings can be different from other authors because of the sample size and different inclusion and exclusion criteria, used by other studies [1–3, 7, 9].

## 5. Conclusions

Advance in medical knowledge and experience facilitates a more detailed analysis of the uterine involution and a longitudinal sonographic study carried out immediately after childbirth is the best way to achieve this. Postpartum ultrasound scan of the uterus is not only safe but also the best way of differential diagnosis of postpartum hemorrhage. The puerperium period after normal labour is dependent on parity. The most intensive uterine involution period is the first month after delivery. The trend of involution in primiparous and multiparous women is similar; however, in multiparous women, it lasts longer than 6–8 weeks. This study speaks for longer duration of physiological uterine size and vascular return from pregnant to nonpregnant state. Also, it is important to apply this approach seeking an early detection of postpartum uterine complications.

## Figures and Tables

**Table 1:** Women's characteristics (mean values ± standard deviation).

	Group I (primiparous)	Group II (multiparous)
Mean age (year)	28.46 ± 2.87	32.36 ± 3.72
Mean body mass index (BMI)	21.04 ± 2.26	22.45 ± 3.66
Mean duration of labour (hours)	8.35 ± 2.88	6.68 ± 2.77
Mean time of the anhydrous period (hours)	6.46 ± 3.91	4.14 ± 2.04
Mean infant birth weight (grams)	3573.96 ± 398.07	3703.64 ± 420.97

**Table 2:** Median characteristics ± standard deviation of the uterine size with minimum and maximum values (mm).

	1st day			30th day			60th day		
	Primiparous	Multiparous	*p*	Primiparous	Multiparous	*p*	Primiparous	Multiparous	*p*
Uterus length (mm)	162.0 ± 17.5 (132–190)	174.0 ± 13.5 (139–190)	0.148	81.0 ± 8.4 (59–91)	83.0 ± 6.7 (68–94)	0.284	63.0 ± 8.2 (49–82)	71.5 ± 12.1 (52–94)	0.069
Uterus width (mm)	119.0 ± 8.7 (36–70)	116.0 ± 12.9 (99–147)	0.805	59.0 ± 6.9 (48–76)	67.0 ± 6.6 (48–74)	0.005	51.0 ± 8.7 (36–70)	53.5 ± 10.7 (39–87)	0.394
AP max (mm)	82.0 ± 10.1 (68–110)	94.0 ± 13.1 (66–116)	0.01	44.0 ± 5.6 (28–54)	48.0 ± 6.7 (32–58)	0.017	36.0 ± 3.9 (30–44)	39.0 ± 8.0 (24–60)	0.059
AP 5 cm below UF (mm)	79.0 ± 10.9 (54–97)	85.0 ± 13.9 (64–116)	0.065	41.0 ± 8.0 (22–51)	40.0 ± 8.5 (17–56)	0.351	22.0 ± 5.2 (15–35)	27.5 ± 9.1 (14–60)	0.015

**Table 3:** Changing of the widest part (median values ± standard deviation) of the uterine cavity (mm).

	1st day	*p*	10th day	*p*	60th day	*p*
Primiparous	10.0 ± 7,8	0.384	9.5 ± 9.9	0.001	4.0 ± 3.9	0.541
Multiparous	13.0 ± 7,4		18.5 ± 7.2		3.0 ± 3.3	

**Table 4:** Changes of RI (resistance index) during puerperium (median values ± standard deviation).

Days	1	3	10	30	42	60
Primiparous	0.54 ± 0,14	0.56 ± 0.10	0.61 ± 0.09	0.69 ± 0.08	0.76 ± 0.09	0.75 ± 0.08
Multiparous	0.63 ± 0,09	0.65 ± 0.08	0.67 ± 0.13	0.75 ± 0.09	0.80 ± 0.07	0.77 ± 0.09
*p*	0.006	0.001	0.004	0.182	0.049	1.000

## Abbreviations

AP: Anteroposterior diameter
RI: Resistance index
UF: Uterine fundus
RPOC: Retained products of conception.
